# *USP25* Expression in Peripheral Blood Mononuclear Cells Is Associated With Bone Mineral Density in Women

**DOI:** 10.3389/fcell.2021.811611

**Published:** 2022-01-24

**Authors:** Jianlin Shen, Bowen Fu, Yanjiao Wu, Yang Yang, Xiaoning Lin, Haibin Lin, Huan Liu, Wenhua Huang

**Affiliations:** ^1^ Guangdong Innovation Platform for Translation of 3D Printing Application, Center for Orthopaedic Surgery, The Third Affiliated Hospital of Southern Medical University, Guangzhou, China; ^2^ Department of Orthopedics, Affiliated Hospital of Putian University, Putian, China; ^3^ Department of Orthopedics, Shunde Hospital of Southern Medical University, Guangzhou, China; ^4^ Guangdong Engineering Research Center for Translation of Medical 3D Printing Application, Guangdong Provincial Key Laboratory of Medical Biomechanics, School of Basic Medical Sciences, Southern Medical University, Guangzhou, China; ^5^ Department of Orthopedics, Affiliated Traditional Chinese Medicine Hospital, Southwest Medical University, Luzhou, China

**Keywords:** osteoporosis, weighted gene coexpression network analysis, menopause, ubiquitination, peripheral blood mononuclear cells, ubiquitin-specific protease 25

## Abstract

Osteoporosis is the most common metabolic bone disease in postmenopausal women. As precursors of osteoclasts, peripheral blood mononuclear cells are accessible and considered suitable models for studying osteoporosis pathology. Ubiquitination is a crucial protein degradation system in bone metabolism. The aim of this study was to identify potential ubiquitination-related genes in PBMCs that are related to osteoporosis pathogenesis. Therefore, we performed an integrated analysis of osteoporosis-related microarray datasets. With the obtained ubiquitination-related gene set, weighted gene coexpression network analysis was performed. The results showed that genes in the turquoise module were correlated with menopause, and 48 genes were identified as hub genes. A differential expression analysis revealed 43 differentially expressed genes between pre- and postmenopausal samples. After integrating the information on differentially expressed menopause-related genes, we found that several members of the ubiquitin-specific protease (*USP*) family (*USP1*, *USP7*, *USP9X*, *USP16*, and *USP25*) were highly expressed in samples from postmenopausal female and that, *USP25* expression was significantly higher in low-BMD samples than in high-BMD samples among samples from premenopausal subjects (*p* = 0.0013) and among all samples (*p* = 0.013). Finally, we verified the protein expression of *USP25* in PBMCs by performing Western blot analysis, which yielded results consistent with the aforementioned results. Moreover, by assessing GTEx datasets, we found that *USP25* expression was highly correlated with *TRAF6* expression in whole blood (*p* < 0.001). We also tested the protein expression levels of TRAF6 in PBMCs and found that it was positively correlated with USP25 expression (*p* = 0.036). Our results reveal that the ubiquitin-specific protease family may play important roles in menopause and that *USP25* is related to osteoporosis pathogenesis.

## 1 Introduction

Osteoporosis (OP) is a systemic bone disease characterized by decreased bone mineral density (BMD) and increased fracture risk (Sambrook and Cooper 2006). Since estrogen profoundly regulates the metabolism of bone cells, postmenopausal osteoporosis (PMOP) with estrogen deficiency is the most typical form of OP (Eastell et al., 2016). Although researchers have analyzed the effects of estrogen on bone metabolism, the mechanism underlying the development of PMOP is still not thoroughly understood (Anagnostis et al., 2021), and comprehensive treatment strategies for PMOP are lacking. Therefore, it is very important to identify the mechanism of PMOP occurrence.

The posttranslational modification mediated by the ubiquitin-proteasome system (UPS) plays very important roles in protein localization, metabolism, regulation and degradation, and is essential for the balance between bone formation and bone resorption (Thibaudeau and Smith 2019; Shen et al., 2021). In addition, the interaction between estrogen and estrogen receptor *a* (ER*α*) can trigger posttranslational modification of ERα through interplay with signaling pathways to promote transcriptional activation and ubiquitin-mediated ER*α* proteolysis (Zhou and Slingerland 2014). However, few studies have investigated the effects of ubiquitination in menopause or PMOP. To date, approximately 2 E1, 35 E2, and more than 600 E3 ubiquitin ligases and hundreds of deubiquitinases have been found in the human ubiquitin system (Heap et al., 2017).

**FIGURE 1:**
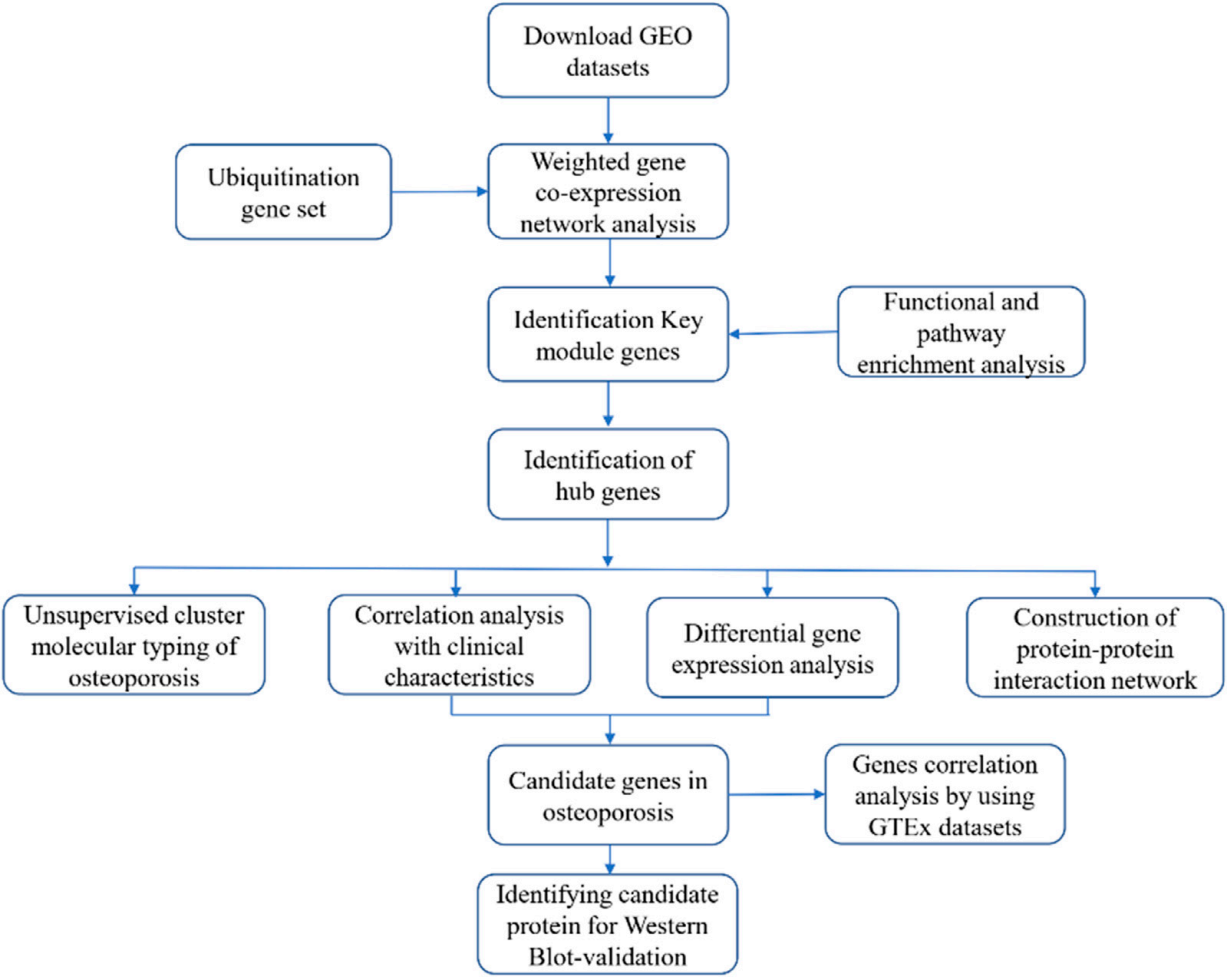
Flowchart of the research

Monocytes, also known as peripheral blood mononuclear cells (PBMCs), are progenitor cells of osteoclasts (Boyle et al., 2003), and can produce cytokines during osteoclastogenesis and bone resorption-related apoptosis (Auffray et al., 2009). Therefore, PBMCs are considered suitable models for studying the pathology of OP (Zhou et al., 2015; Zhang et al., 2016), and a genomic analysis of PBMCs may reveal genes in osteoclast progenitor cells that are involved in OP (Pietschmann et al., 2001). In this study, we used weighted gene coexpression network analysis (WGCNA) to analyze microarray data (data from 80 monocyte samples in the Gene Expression Omnibus [GEO] database) of PBMCs in pre- and postmenopausal females with low or high BMD to characterize ubiquitination genes associated with menopause and BMD. The results indicated that ubiquitin-specific protease 25 (*USP25*) may be an important ubiquitination gene in menopause and OP. Figure 1.

## 2 Materials and Methods

### 2.1 Data Collection and Preprocessing

(1) GEO data downloading and processing: The microarray dataset GSE56815 was downloaded from the GEO database (https://www.ncbi.nlm.nih.gov/geo/) (Edgar et al., 2002); details are provided in Table 1. The data were preprocessed as follows: The downloaded dataset includes observations of log2-transformed quantile-normalized signal intensity. First, the probes were mapped to genes, and empty probes were removed. When multiple probes corresponded to the same gene, we considered the median expression level to be the expression level of the gene.
(2) Ubiquitination-related gene set characteristics: The ubiquitination-related gene set was downloaded from the Integrated Annotations for Ubiquitin and Ubiquitin-like Conjugation Database (iUUCD) database (http://iuucd.biocuckoo.org/), and it included genes encoding 27 E1, 109 E2, and 1153 E3 ubiquitin ligases, 164 deubiquitinating enzymes (DUBs); 396 ubiquitin-binding domains (UBDs); and 183 ubiquitin-like domains (ULDs) from multiple species. A total of 806 human gene symbols are reported in the database. A total of 574 genes were found in the GSE56815 dataset, and this gene set was subsequently used for WGCNA.

**TABLE 1:** mRNA expression profile dataset in the GEO database.

Accession number	Race	Premenopausal female		Postmenopausal female		Tissue/cell
		High BMD	Low BMD	High BMD	Low BMD	
GSE56815	Caucasian	20	20	20	20	Monocytes

**TABLE 2:** Population characteristics of subjects for USP25 protein comparisons. Values are medians (interquartile ranges).

	Premenopausal females		Postmenopausal females	
	Low BMD (Z<=−2.0)	High BMD (Z>=−1.0)	Low BMD (T<=−2.5)	High BMD (T>=−1.0)
Number	6	5	6	6
Age (years)	38 (36–42)	40 (36–49)	56 (52–67)	56 (54–59)
BMI (kg/m^2^)	21.66 (17.36–27.06)	21.99 (19.63–24.03)	22.58 (19.95–24.97)	25.16 (22.76–28.3)

### 2.2 Construction of a Coexpression Network Based on Ubiquitination-Related Genes

The GSE56815 dataset was used to construct a gene coexpression module network with the R software package “WGCNA” (Langfelder and Horvath 2008). For pairwise genes analysis, Pearson’s correlation matrices were initially constructed, and then, an adjacency matrix was constructed on the basis of the power function correlation between two genes. The formula was α*ij* =|cor (x_*i*_,x_*j*_) |^*β*^ (where cor (x_*i*_,x_*j*_) =Pearson’s correlation coefficient between gene i and gene j, and aij = adjacency between gene i and gene j). After selecting the optimal *ß* value, the adjacency matrix was transformed into a topological overlap matrix (TOM). Using the TOM, we performed average-linkage hierarchical clustering to cluster genes, and we set the minimum number of genes for each gene network module to 30 on the basis of the standard of a hybrid dynamic shearing tree. Each module was analyzed by calculating the dissimilarity of module eigengenes, and the modules that were close to each other were merged to form new modules.

### 2.3 Functional Enrichment Analysis of Genes in Key Modules

Gene Ontology (GO) and Kyoto Encyclopedia of Genes and Genomes (KEGG) enrichment analyses were performed by using the R package cluster Profiler, and the 20 most enriched genes and pathways were visualized in a bubble chart. (In cases with fewer than 20 enriched pathways, all pathways were shown in the chart).

### 2.4 Identification of Clinical Phenotype-Significant Modules and Hub Genes

We performed a module-trait relationship analysis with the genes identified by WGCNA to estimate the correlations between modules and phenotypes. Gene significance (GS), defined as the correlation between gene expression and phenotype (here, BMD and menopausal status) and module membership (MM), defined as the correlation between gene expression and the eigengenes in the module of interest, were used to generate scatter plots. The module most related to clinical phenotype was selected for follow-up analysis. Additionally, genes with MM ≥ 0.7 and GS > 0.2 were identified as hub genes in phenotype-significant modules.

### 2.5 Differential Gene Expression Analysis

Using the GSE56185 dataset, we compared hub genes in high-BMD and low-BMD samples. Correlations between hub genes and a clinical characteristic (menopause) were also analyzed. Differential gene expression was analyzed by using R language software, and *p* < 0.05 was set as the criterion for statistical significance. Normally distributed data were analyzed by unpaired t tests, and nonnormally distributed data were analyzed by Mann–Whitney U tests. The ggpubr package of R was used to construct histograms.

### 2.6 Construction of a Protein-Protein Interaction Network

A hub gene PPI network was constructed based on the STRING database (https://string-db.org/), which includes protein interactions, by using the R package STRINGdb. Cytoscape software was used to visualize the network.

### 2.7 Unsupervised Cluster Molecular Typing in Osteoporosis Based on Hub Genes

The R package factoextra was used to evaluate various clustering trees and display them graphically. The k-means clustering method was used for sample classification, and the within-cluster sum of squares (wss) elbow rule was used to determine the optimal number of clusters, the R package factoextra was used to visualize the clustering results, and the R package pheatmap was used to generate a heatmap.

### 2.8 Identifying Candidate Proteins for Western Blot Validation Subjects

We collected data on 23 females who had undergone health checkups at the Affiliated Hospital of Putian University and divided the subjects into 4 groups (Table 1): premenopausal with low BMD (Z<=−2.0, *n* = 6), premenopausal with high BMD (Z>=−1.0, *n* = 5), postmenopausal with low BMD (T<=−2.5, *n* = 6) and postmenopausal with high BMD (T>=−1.0, *n* = 6). Premenopausal females with abnormal hormone levels were excluded.

#### (1) Peripheral Blood Mononuclear Cells Isolation

We isolated PBMCs by density gradient centrifugation. We carefully overlaid diluted blood over a Ficoll layer (Solarbio, Beijing, China) by resting the pipette tip containing the blood against the wall of the conical tube containing the Ficoll. After centrifugation, a fluffy white layer of PBMCs at the interphase was collected.

#### (2) Western Blot Analysis

Total protein was extracted with RIPA lysis buffer (ABclonal, Wuhan, China), and quantified by bicinchoninic acid (BCA) assay. After sample preparation, protein samples (20 μg) were separated by electrophoresis on a 10% SDS-PAGE gel and transferred to a PVDF membrane. After blocking with 5% nonfat milk in TBST for 2 h at room temperature, the membrane was incubated overnight at 4 C with the following primary antibodies: anti-USP25 (diluted 1:1,000, A7975, ABclonal, Wuhan, China), anti-TRAF6 (diluted 1:500, D21G3, Cell Signaling Technology, Boston, United States) and anti-GAPDH (diluted 1:10,000, ab181602, Abcam, Cambridge, United Kingdom). The membrane was then incubated with secondary antibody (anti-rabbit, 1:10,000, BS13278, Bioworld, Minnesota, United States) for 2 h at room temperature. After washing three times in TBST, protein bands were visualized with enhanced chemiluminescence (ECL) reagent (ABclonal, Wuhan, China). The gray values of the protein bands were quantified with ImageJ software (ImageJ 1.53, NIH, United States). Samples with poor expression of GAPDH were excluded, and the results were statistically evaluated by Student’s *t* test. Differences with a *p* value <0.05 were considered statistically significant.

#### (3) Correlation Between *USP25* and *TRAF6* Expression in Whole Blood as Indicated by GTEx Dataset Analysis

Expression datasets obtained from GTEx (http://gepia.cancer-pku.cn/index.html) were used to analyze the correlation between *USP25* and *TRAF6* expression in whole blood. We used the nonlog scale for calculation and the log-scale axis for visualization.

## 3 Results

### 3.1 Construction of a Coexpression Network Based on Ubiquitinated Genes

**FIGURE 2:**
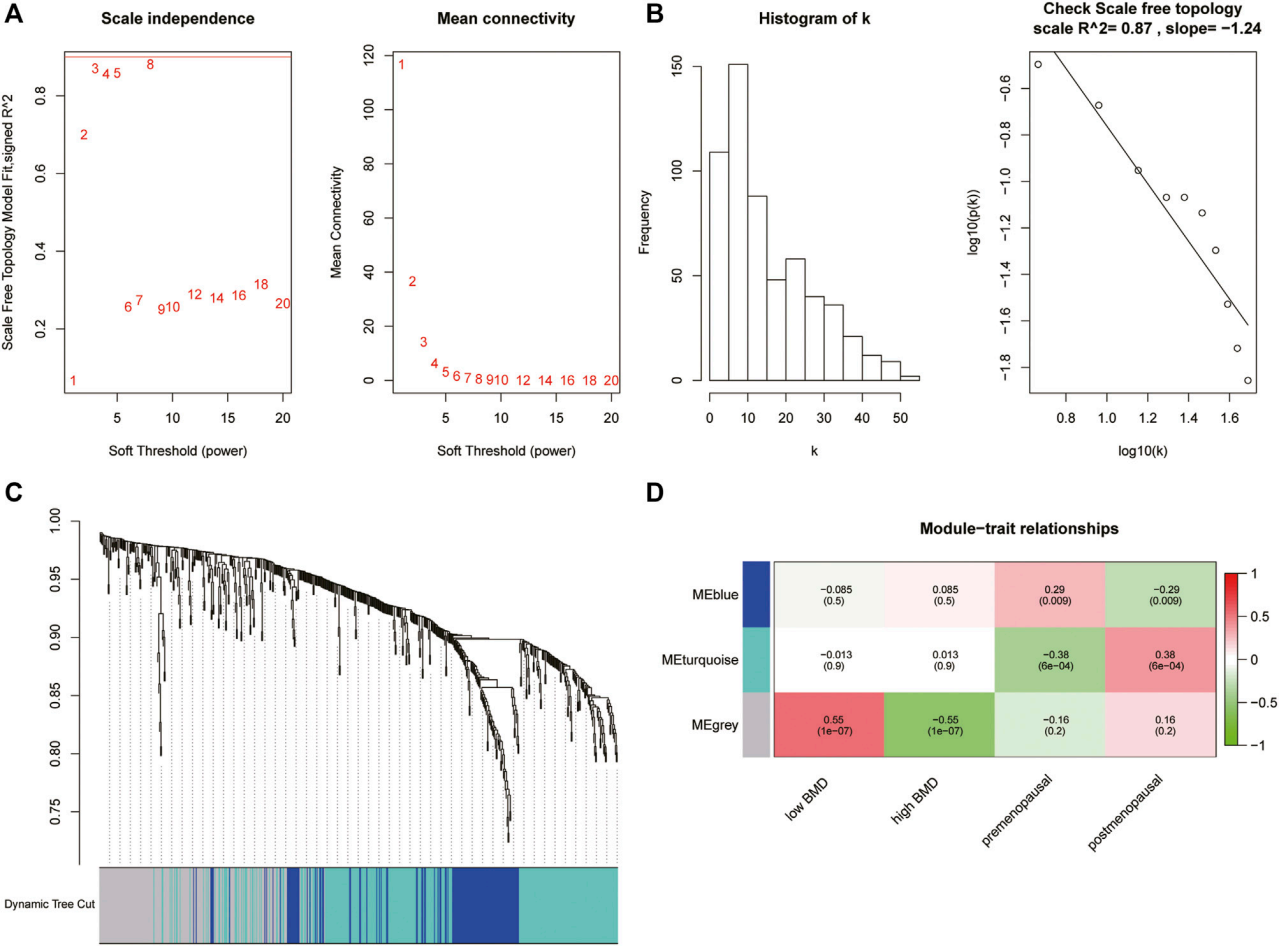
**(A, B)** Diagrams showing the network topology analysis based on soft thresholding. **(C)** Results of dynamic tree cutting based on the topological overlap matrix (TOM). **(D)** Heatmap showing the correlation between the module eigenvalue and menopausal phenotype or BMD phenotype.

The results showed that the log(k) of a node with connection degree k was negatively correlated with the log (P(k)) of node probability with a correlation coefficient greater than 0.8, indicating that the coexpression network conformed to a scale-free network. For the GSE56815 data (80 samples), we chose the optimal *ß* value of 3 (Figure 2A), and a total of 3 modules were obtained (Figure 2D): a gray module containing 160 genes, a turquoise module containing 284 genes, and a blue module containing 130 genes. The turquoise module was selected as the key module for subsequent analysis.

### 3.2 Functional Enrichment Analysis of Genes in Key Modules

**FIGURE 3:**
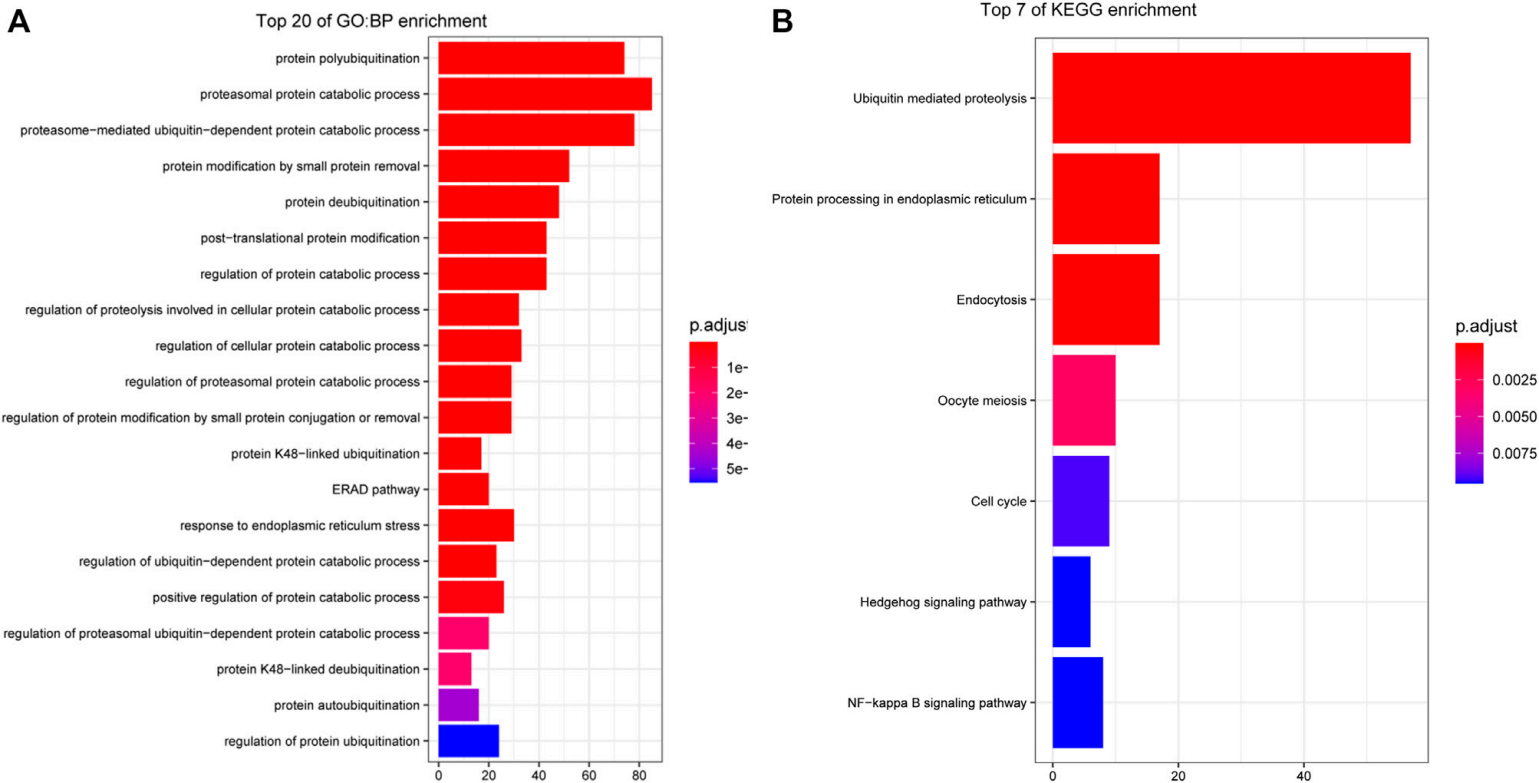
Functional enrichment analysis of genes in key modules. **(A)** GO biological process (BP) enrichment: The three most enriched BP terms are protein polyubiquitination, proteasome protein catabolic process, and proteasome-mediated ubiquitin-dependent catabolic process. **(B)** KEGG pathway enrichment: The three most enriched pathways are ubiquitin-mediated proteolysis, protein processing in the endoplasmic reticulum, and endocytosis.

We used the R package cluster Profiler to perform GO and KEGG enrichment analysis with the 284 genes in the turquoise module and used the R package enrich plot to visualize the 20 most enriched pathways in a histogram according to, p. adjust<0.05. The GO biological process terms are shown in Figure 3A, and the KEGG pathways are shown in Figure 3B.

### 3.3 Identification of Clinical Phenotype-Significant Modules and Hub Genes

**FIGURE 4:**
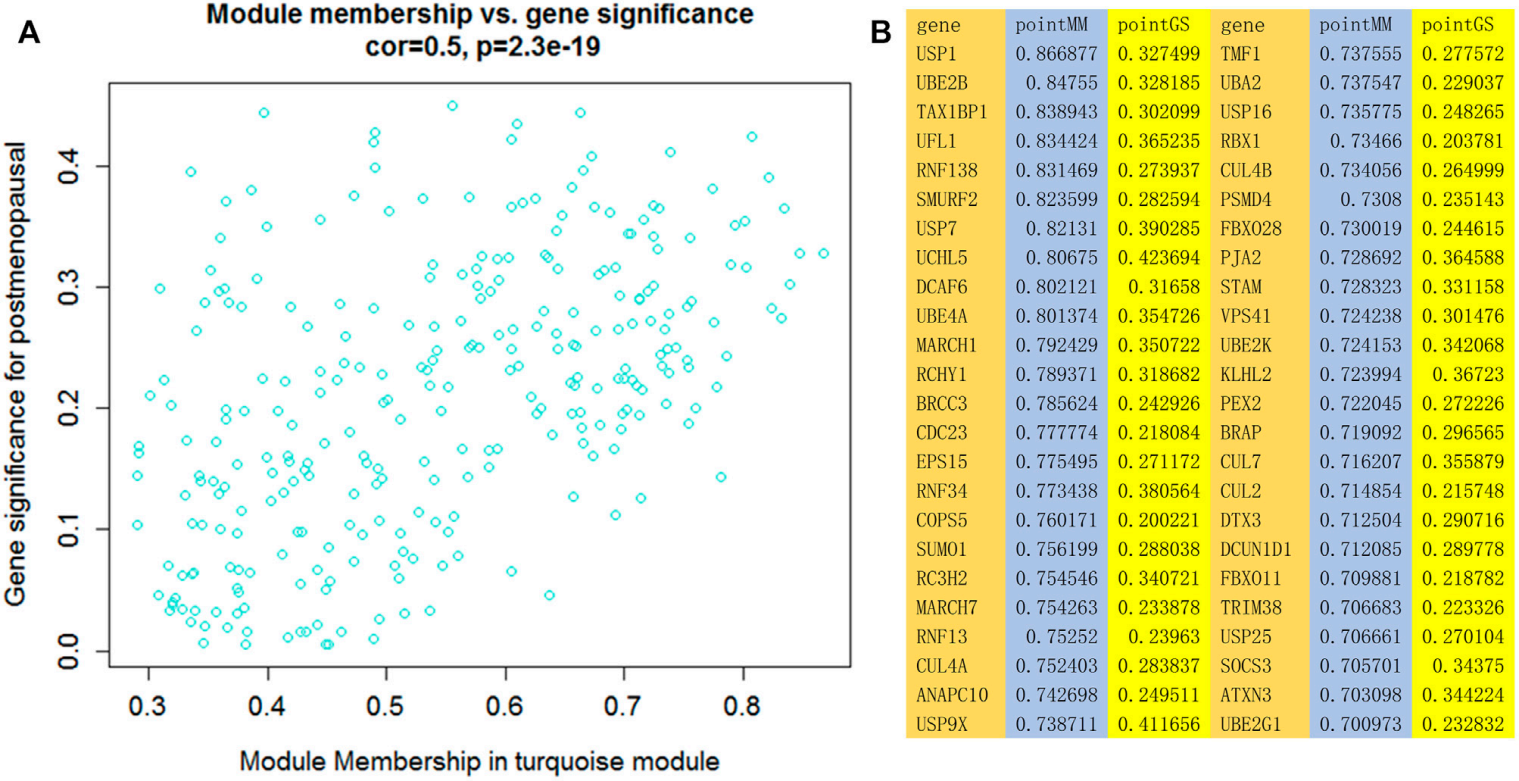
**(A)** Scatter plot of module membership (MM) and gene significance (GS) in the turquoise module. A negative correlation between MM and GS was observed in this module. **(B)** Genes with MM ≥ 0.7 and GS > 0.2.

We generated scatter plots of module members (MMs) in the turquoise module and calculated GS with the data on postmenopausal women. In the turquoise module, a positive correlation was observed between MM and GS, with a correlation coefficient of 0.5 and *p* < 0.05. Additionally, 48 genes with MM ≥ 0.7 and GS > 0.2 were identified as hub genes in phenotype-significant modules. Figure 4.

### 3.4 Differential Gene Expression Analysis

**FIGURE 5:**
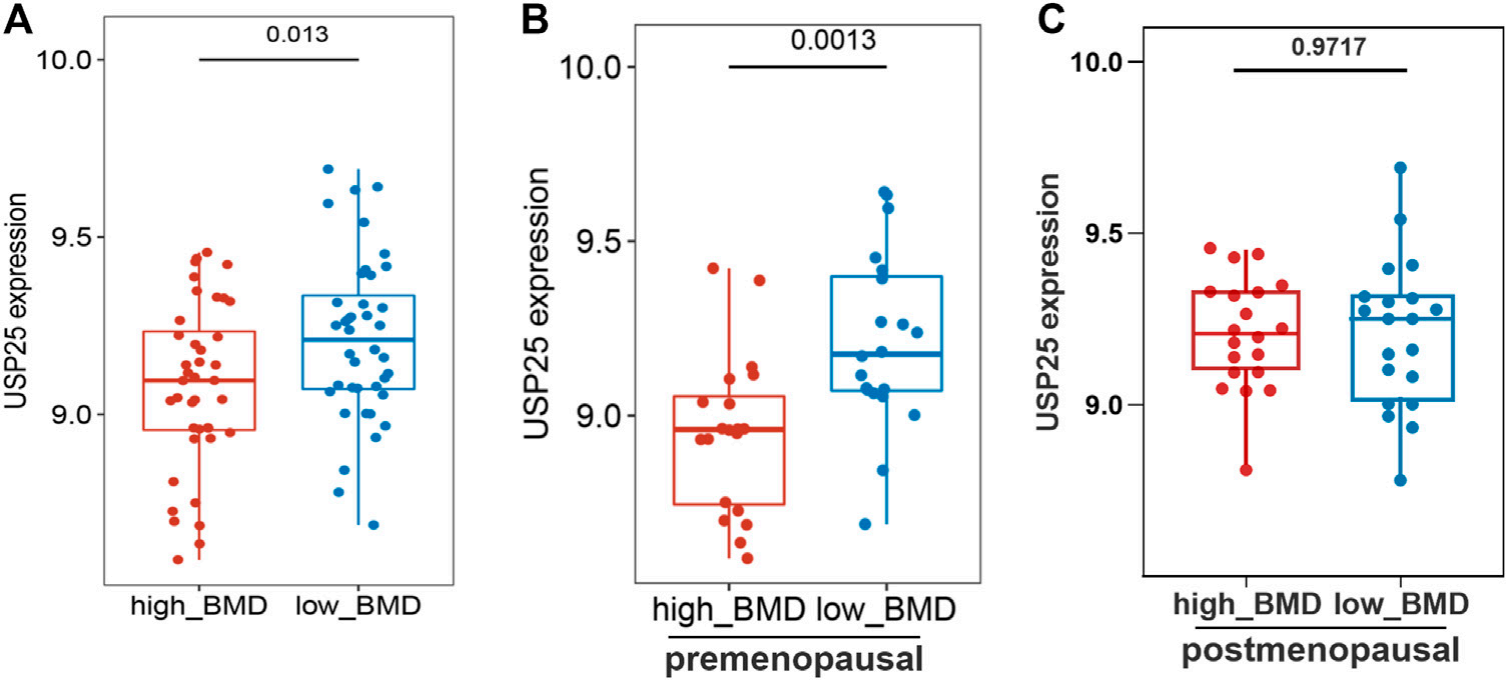
Correlation analysis of hub genes and clinical indicators (high and low density) in the turquoise module. **(A)** Differential gene expression of USP25 in all samples. **(B)** Differential gene expression of USP25 in premenopausal samples. **(C)** Differential gene expression of USP25 in postmenopausal samples.

The differential hub gene expression analysis of high- and low-BMD samples indicated that *USP25* expression was significantly higher in the low-BMD samples (Figure 5A). In addition, the analysis of pre- and postmenopausal samples showed that the expression of *USP25* was significantly correlated with BMD in the premenopausal samples (Figure 5B) but not in the postmenopausal samples (Figure 5C).

### 3.5 Correlation Analysis of Hub Genes and Clinical Characteristics

**FIGURE 6:**
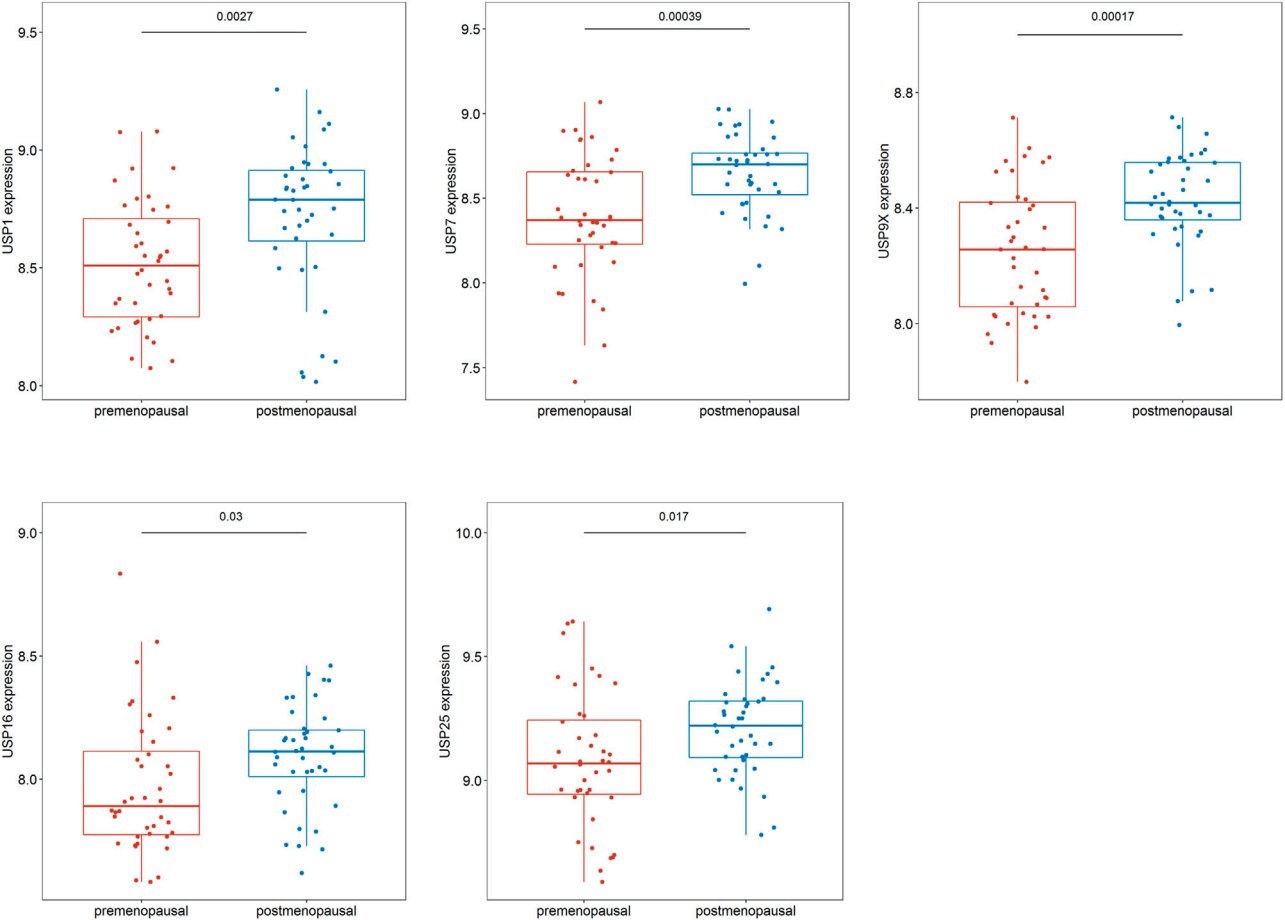
Differences in hub gene expression in the pre- and postmenopausal samples.

We identified genes with MM ≥ 0.7 and GS > 0.2 in the turquoise module as hub genes. We used R language software to analyze the 48 hub genes in the pre- and postmenopausal groups and found that 43 genes exhibited significantly different expression between the pre- and postmenopausal samples (Supplementary Figure S1). Among these genes, several genes encoding ubiquitin-specific peptidases (*USP1, USP7, USP9X, USP16,* and *USP25*) were found to exhibit significantly increased expression in the postmenopausal samples (Figure 6).

### 3.6 Construction of a Protein-Protein Interaction Network

**FIGURE 7:**
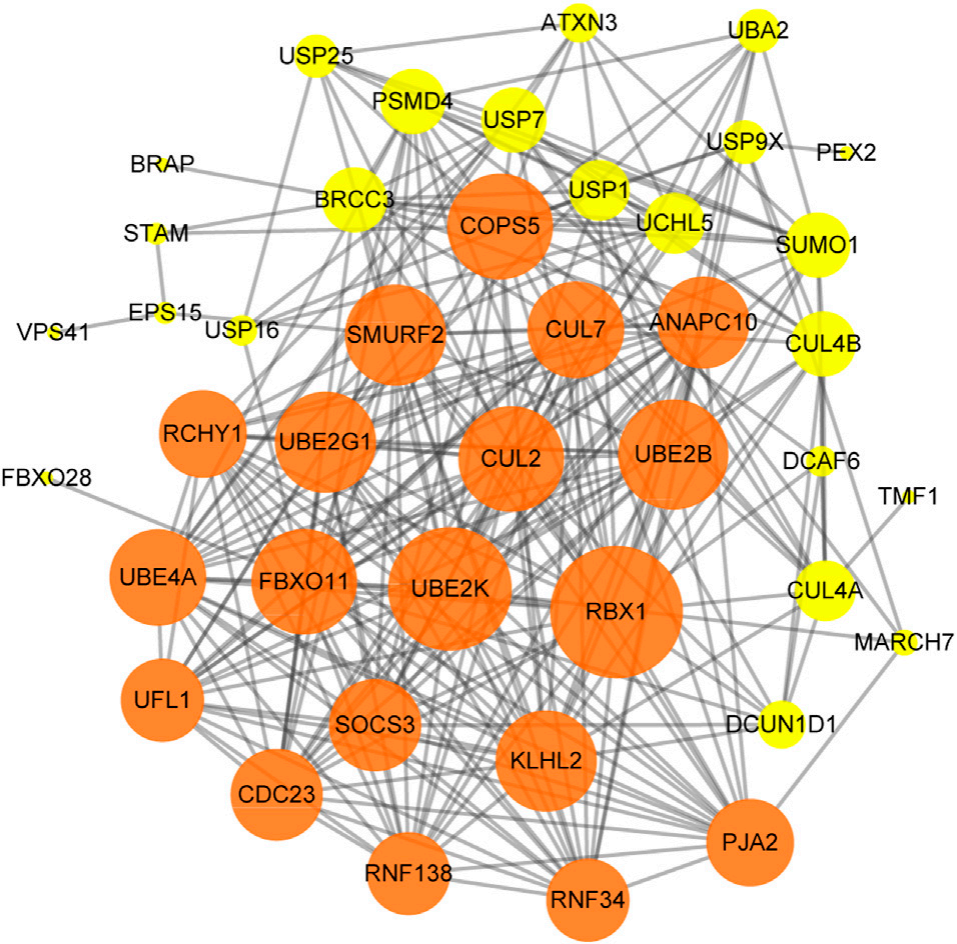
The Protein-Protein interaction (PPI) network of the hub genes in the turquoise module. Nodes represent proteins, and edges represent interactions between proteins.

The PPI network of hub genes in the turquoise module was established by Cytoscape software. We set a minimum interaction score of 400 to filter interaction pairs and retain proteins with interaction pairs >1. The “high degree” nodes in the PPI network were defined as “hub proteins,” and the degrees were defined by the number of neighbors directly connected to the node. The green nodes represented central nodes and had more than 15 connections, and the yellow nodes represented central nodes and had 1–15 connections. The significant hub proteins included *RBX1* (degree = 28), *UBE2K* (degree = 26) and *UBE2B* (degree = 23). *USP25* was connected to *SMURF2, USP16, USP1, USP7, SUM O 1, CUL2* and *ATXN3* (Figure 7).

### 3.7 Unsupervised Cluster Molecular Typing of Osteoporosis Based on Hub Genes

**FIGURE 8:**
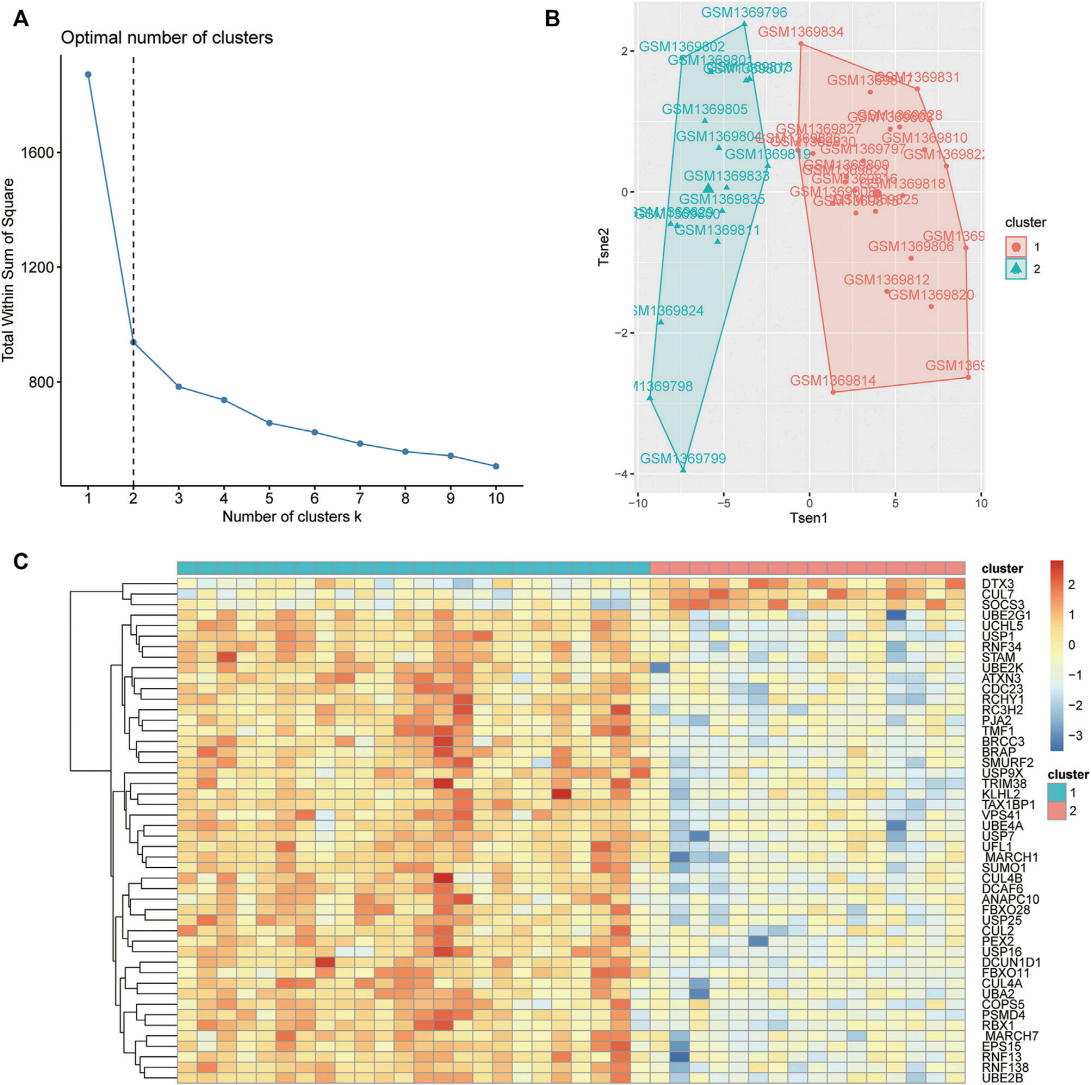
Unsupervised cluster molecular typing of OP. **(A)** Optimal number of clusters based on the error sum of squares (SSE). **(B)** T-SNE diagram showing sample clustering. **(C)** Heatmaps showing the expression of hub genes.

According to the results of the WGCNA of the coexpression modules, the OP (low-density) samples were classified by the k-means clustering method based on the 48 hub genes in the green module, and the wss elbow rule was used to determine the optimal number of clusters. As shown in Figure 8A, wss was negligibly reduced after *K* = 2; therefore, *K* = 2 was selected as the number of clusters. Then, the R package factoextra was used to reduce the dimensionality of the gene expression data. As shown in Figure 8B, the samples could be clearly classified into 2 categories. Finally, the R package pheatmap was used to generate heatmaps of the expression of the 48 hub genes in cluster 1 and cluster 2.

### 3.8 Identifying Candidate Proteins for Western Blot Validation

**FIGURE 9:**
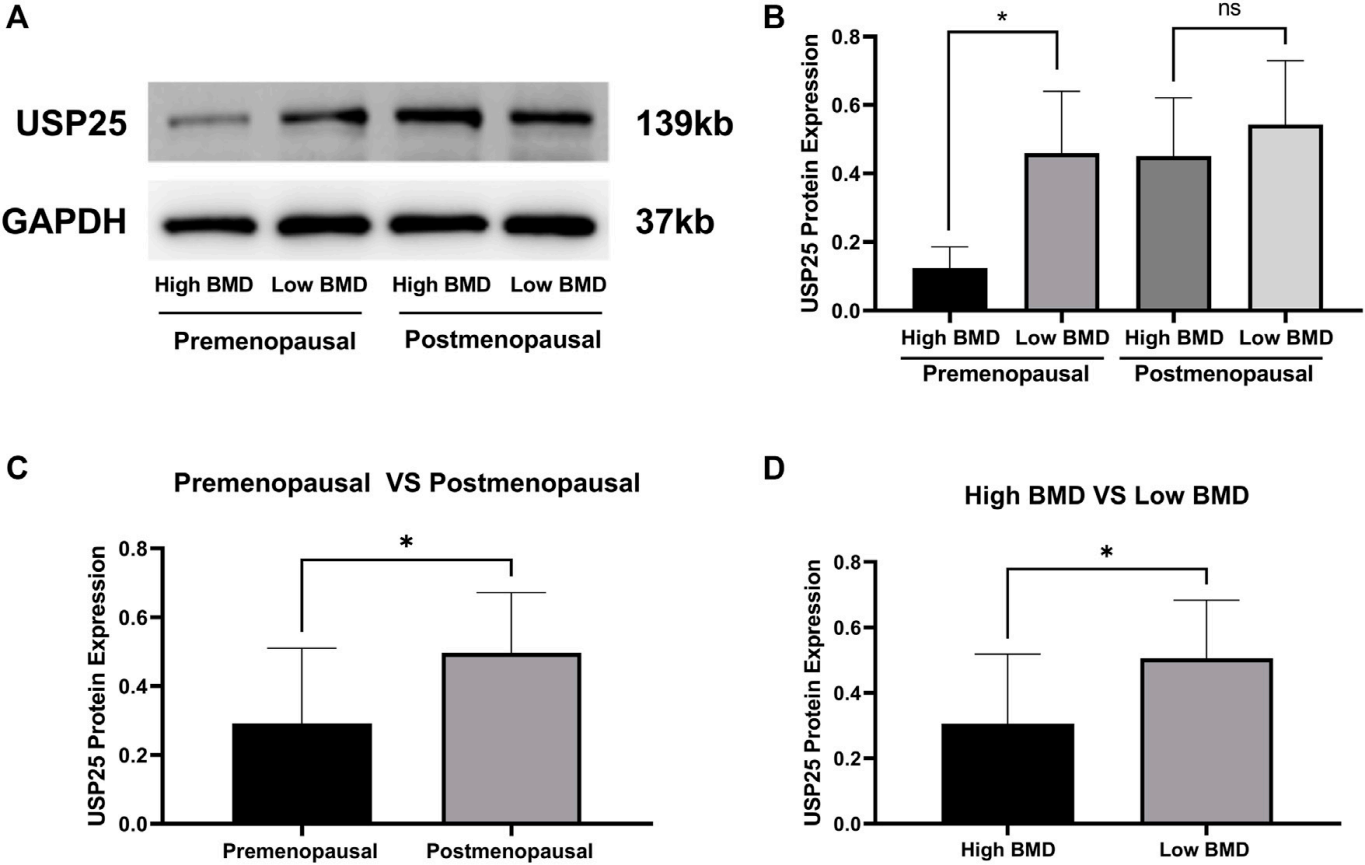
Western blot results validated the protein expression of *USP25* in PBMCs. **(A and B)** *USP25* protein expression in pre- and postmenopausal females with different BMD levels. **(C)** Differences in *USP25* protein expression level between pre- and postmenopausal females. **(D)** Differences in *USP25* protein expression level between high-BMD and low-BMD samples. The data are reported as the means ± SD, *p < 0.05.

Consistent with the aforementioned results, the protein expression of *USP25* in PBMCs was significantly higher in low-BMD samples than in high-BMD samples both among premenopausal females and among all samples, but *USP25* expression was not significantly different between the high- and low-BMD samples from postmenopausal females (Figures 9A,B,D). Expression of the *USP25* protein was significantly higher in the samples from postmenopausal females than in those from premenopausal females (Figure 9C).

### 3.9 Correlation Between USP25 and TRAF6 Expression in Whole Blood as Indicated by GTEx Dataset Analysis

**FIGURE 10:**
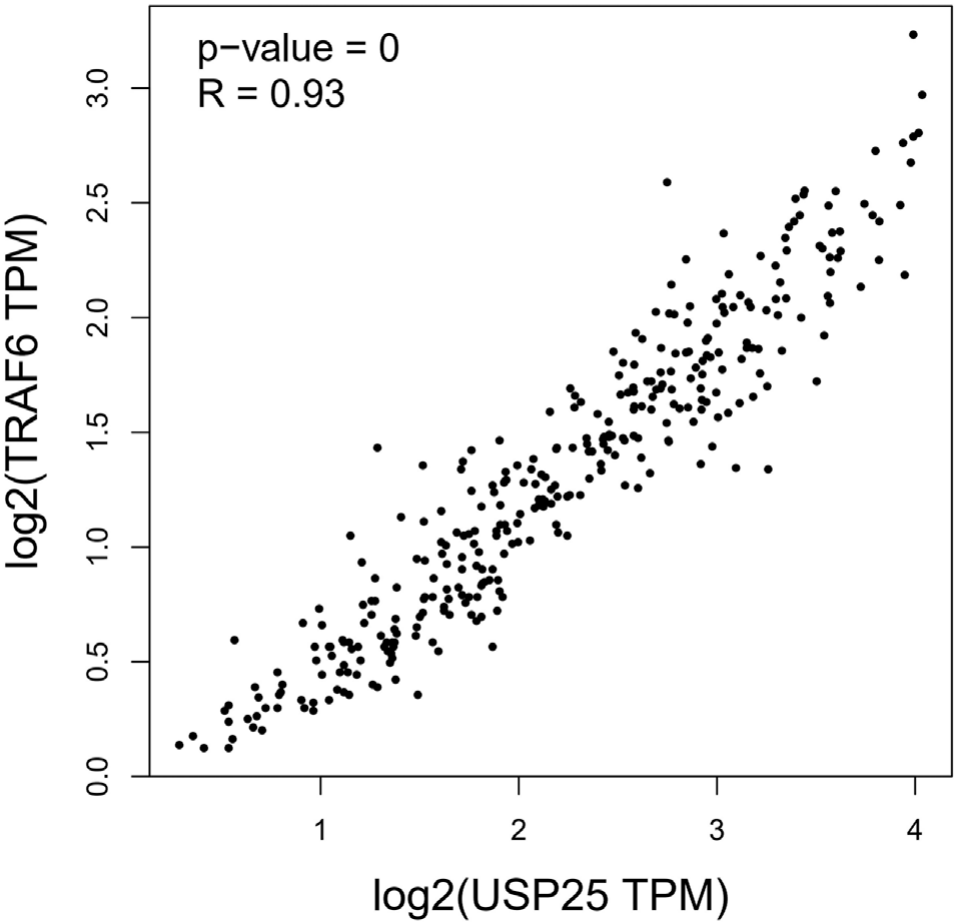
Correlation between *USP25* and *TRAF6* expression in whole blood.

Previous studies have shown that the *TRAF6* protein may be a substrate of *USP25*(B. Zhong et al., 2012). Therefore, we used GTEx expression datasets to analyze the correlation between *USP25* and *TRAF6* expression in whole blood. The results showed that in whole blood, the expression of *USP25* was highly positively correlated with the expression of *TRAF6* (R = 0.93, *p* < 0.001). Figure 10.

### 3.10 Correlation Between Protein Expression of USP25 and TRAF6 in Peripheral Blood Mononuclear Cells

We also tested the protein expression of TRAF6 in PBMCs and found that it was positively correlated with USP25 expression (*p* = 0.036).

## 4 Discussion

PMOP accounts for a large proportion of OP cases and seriously affects the health and quality of life of elderly individuals. However, the mechanism that triggers PMOP has not yet been thoroughly elucidated, and comprehensive treatment strategies are lacking. Circulating monocytes are precursors of osteoclasts and are essential for bone destruction and remodeling (Xiao et al., 2019). Previous studies have revealed large differences in ubiquitination-related gene expression in OP between pre- and post-menopause (Yang et al., 2019). In this study, we used WGCNA to assess the microarray data of PBMCs and characterize ubiquitination-related genes associated with menopause and BMD. We found that several genes encoding ubiquitin-specific peptidases (*USP1, USP7, USP9X, USP16*, and *USP25* in the USP family) play important roles in menopause, and that, among these genes, USP25 is associated with the occurrence of OP.

By performing a GO analysis, we found that hub genes were significantly enriched in the GO biological process term of protein deubiquitination. Ubiquitination and deubiquitination have emerged as critical posttranslational regulators of homeostasis and intracellular protein functions (Thibaudeau and Smith 2019; Hariri et al., 2021). The human genome encodes approximately 100 DUBs, of which the USP family, comprising approximately 58 different members, forms the largest cluster (Nijman et al., 2005). USPs have received extensive attention due to their distribution and functional diversity in eukaryotic tissues and organs. Previous studies have demonstrated the critical roles of USPs in bone metabolism. For example, several USPs, including *USP1* (Williams et al., 2011), *Usp2* (Shirakawa et al., 2016), *USP4* (F. Zhou et al., 2016), *USP7* (Ji et al., 2019), *USP15* (Herhaus et al., 2014), *USP34* (Guo et al., 2018) and *Usp53* (Hariri et al., 2021), have been suggested to be involved in osteogenic differentiation and bone formation. However, the roles of USPs in the regulation of osteoclastogenesis and bone resorption have not been extensively studied.

The regulatory role of *USP25* in OP is unclear. To investigate the involvement of *USP25* in BMD regulation, we analyzed KEGG pathways and found that the NF-κ B signaling pathway was significantly enriched. *TRAF6* is an E3 ubiquitin ligase that can catalyze Lys63-linked ubiquitination, which is essential for transducing *RANKL* signaling and activating downstream NF-κB signaling pathways (Liao et al., 2019). Zhong et al. showed that *TRAF6* is hyperubiquitinated in *USP25*-deficient cells, which indicated that *USP25* inhibits the physiological ubiquitination of *TRAF6* (Zhong et al., 2012). Through its DUB activity, *USP25* binds to *TRAF6* to prevent its ubiquitination and degradation (Zhong et al., 2013; Lin et al., 2015). *Act1* is an E3 ligase located upstream of *TRAF6* that can construct K63 ubiquitin chains on *TRAF6*, inducing its degradation (Liu et al., 2009). *USP25* might directly cleave *Act1*-mediated K63 polyubiquitin chains from *TRAF6* without interfering with the E3 ligase activity of *TRAF6* itself (Ma 2012; Zhong et al., 2012). To confirm the correlation between *USP25* and *TRAF6* in peripheral blood, we analyzed the expression data obtained from whole blood donated by healthy patients in the GTEx database. The results showed that the expression level of *USP25* was highly positively correlated with the expression level of *TRAF6*. We also tested the protein expression levels of TRAF6 in PBMCs and found that it was positively correlated with USP25 expression. Moreover, monocytes are the progenitor cells of osteoclasts. Therefore, we concluded that *USP25* may participate in the differentiation of osteoclasts by stabilizing *TRAF6* expression.

**FIGURE 11:**
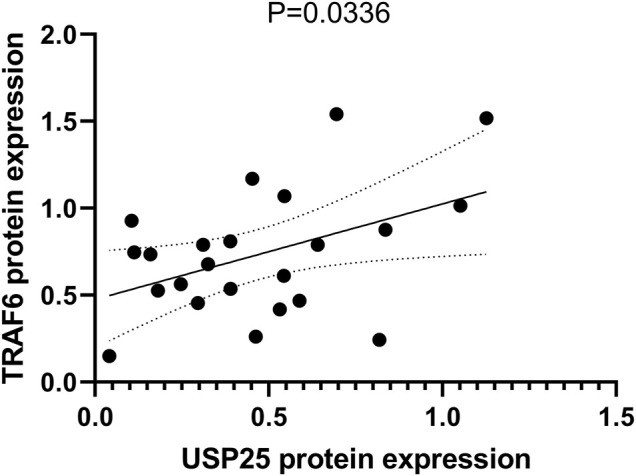
Correlation between protein expression of *USP25* and *TRAF6* in PBMCs.

**FIGURE 12:**
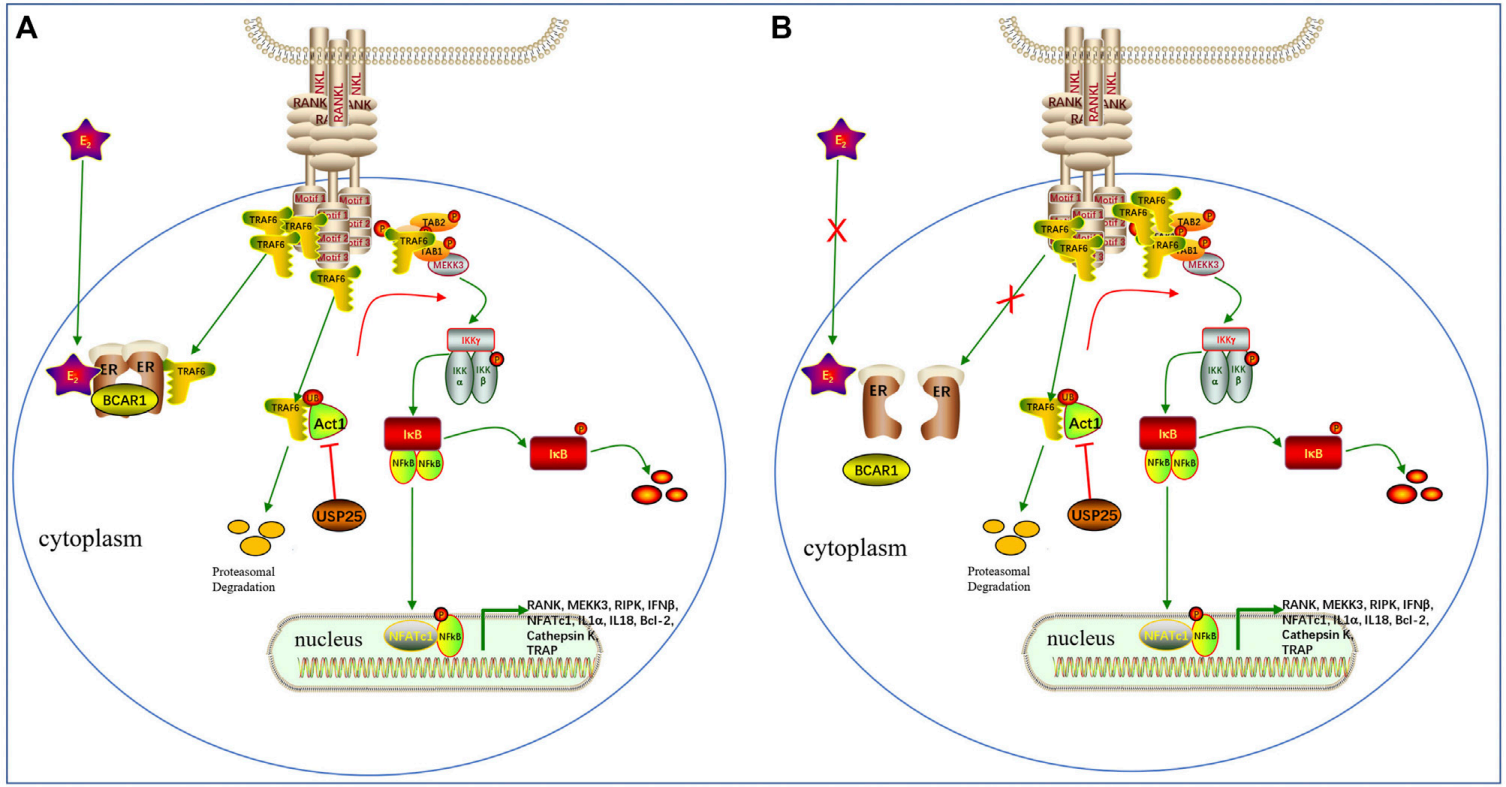
The potential role of USP25 in the effects of estrogen on RANKL signaling **(A)**. Before menopause, estrogen and RANKLpromote the binding ofERα, BCAR1and TRAF6and inhibit the TRAF6protein interactions necessary for formation of the Tab 1-Tab 2-Tak1complex upstream of NF-κB.The remaining free TRAF6 protein forms a complex with Tab 1-Tab 2-Tak1 and activatesdownstream NF-κB to maintain normal bone turnover.USP25can deubiquitinate and stabilize the TRAF6protein, thereby promoting downstream NF-κBactivation.**(B)**. After menopause, the lack of estrogen increases the amount of unbound TRAF6protein, which leads to increased nuclear localization and activation of NF-κB, thereby promoting the formation of osteoclasts. In addition, the protein expression of USP25, which stabilizes TRAF6, and the expression of TRAF6are simultaneously upregulated.However, USP25 no longer plays an influential role in osteoclast differentiation after menopause.

Estrogen has been implicated in the regulation of *RANKL* signaling and inhibits bone resorption in premenopausal women; however, the underlying mechanism has not yet been fully elucidated (Anagnostis et al., 2021). In addition, it has also been proposed that estrogen may regulate bone resorption through its direct effect on osteoclasts, which may be mediated, at least in part, by fast-acting nongenomic mechanisms (Kousteni et al., 2002; Saintier et al., 2006). Robinson LJ et al. found that estrogen inhibited *RANKL*-stimulated osteoclastic differentiation of human monocytes through estrogen and *RANKL*-regulated interaction of *ER-α* with *BCAR1* and *TRAF6* (Robinson et al., 2009). In this study, we found that the expression of *USP25* was correlated with BMD only in the samples from premenopausal females, not in those from postmenopausal females. A possible explanation for this finding may be related to the fact that *TRAF6* bind with *ERα* and *BCAR1* mostly in premenopausal females due to the presence of estrogen (Robinson et al., 2009). In premenopausal females, *USP25* regulates the remaining free *TRAF6* and promotes the differentiation of monocytes into osteoclasts. However, in postmenopausal females, the lack of estrogen causes the release of a large amount of *TRAF6*, stimulating the upregulation of *USP25* and ultimately promoting osteoclast differentiation. However, in these women, *USP25* does not play an influential role in osteoclast differentiation (Figure 11,12).

OP has been divided into two broad types: primary OP and secondary OP. Primary OP includes degenerative OP, PMOP, and idiopathic OP, and secondary OP is a condition related to weakened bone due to other health disorders. In our study, we categorized low-BMD samples into 2 categories according to the expression of 48 hub genes, and significant differences were found in the expression of *USP16* and *CUL4A* between the two clusters (Supplementary Figure S2). This new classification method for OP based on ubiquitination-related genes may be the focus of future studies.

In summary, in this study, we performed WGCNA to construct a gene coexpression network to identify and validate ubiquitination-related genes associated with menopausal status and BMD. Subsequently, *USP25* was found and subsequently confirmed to be related to the occurrence of OP. These results identify *USP25* as a possible therapeutic target for PMOP, and future research will focus on the possible mechanism of *UPS25* involvement in the later period of OP.

## Data Availability

The datasets presented in this study can be found in online repositories. The names of the repository/repositories and accession number(s) can be found below: https://www.ncbi.nlm.nih.gov/geo/, GSE56815.
