# Supplementary figures and images for: USP25 Expression in Peripheral Blood Mononuclear Cells Is Associated With Bone Mineral Density in Women
Source: Front Cell Dev Biol. 2022 Jan 24;9:811611. doi: 10.3389/fcell.2021.811611 (PMC8819182; doi:10.3389/fcell.2021.811611)

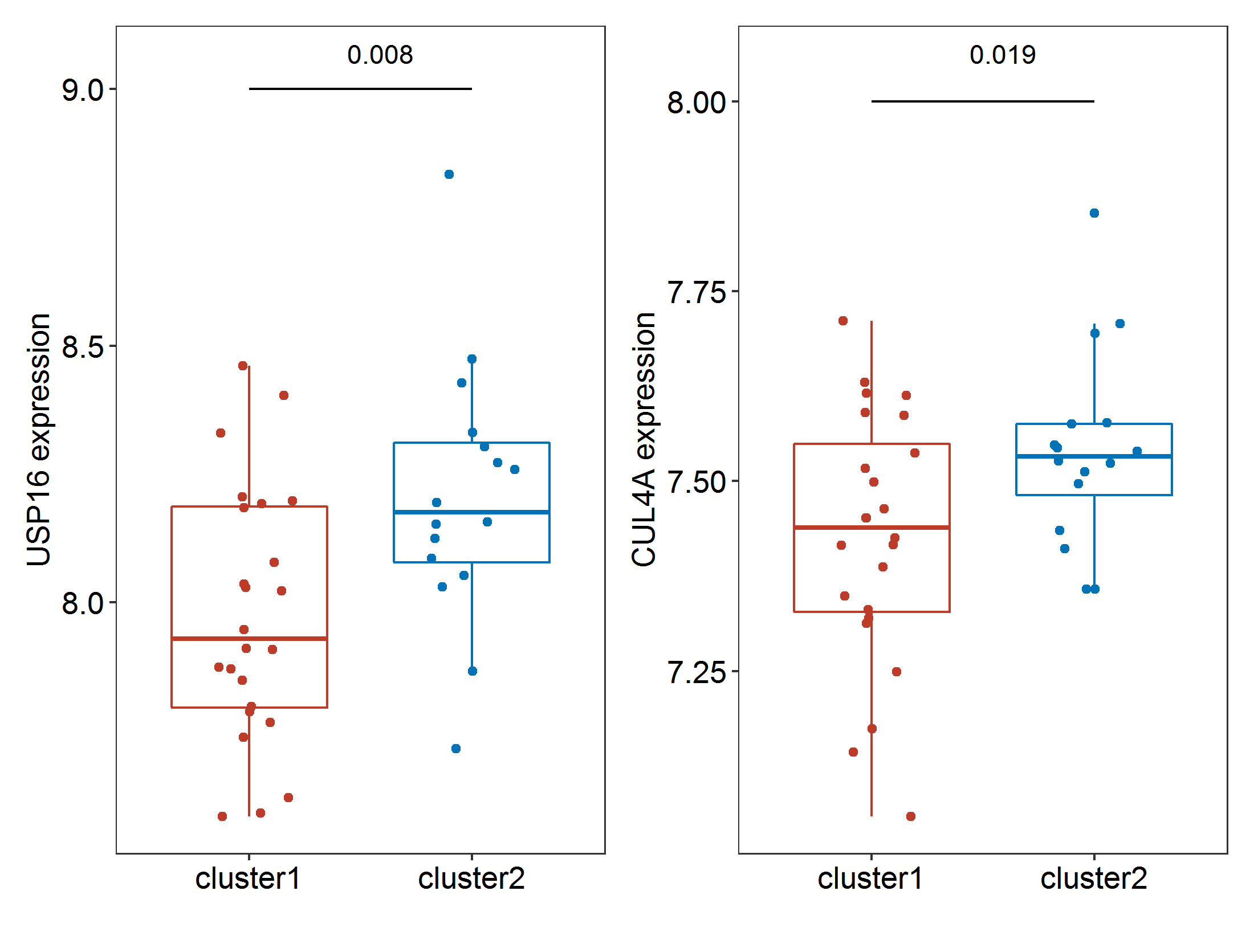

Supplement: Supplementary file 1 [file Image1.TIFF]

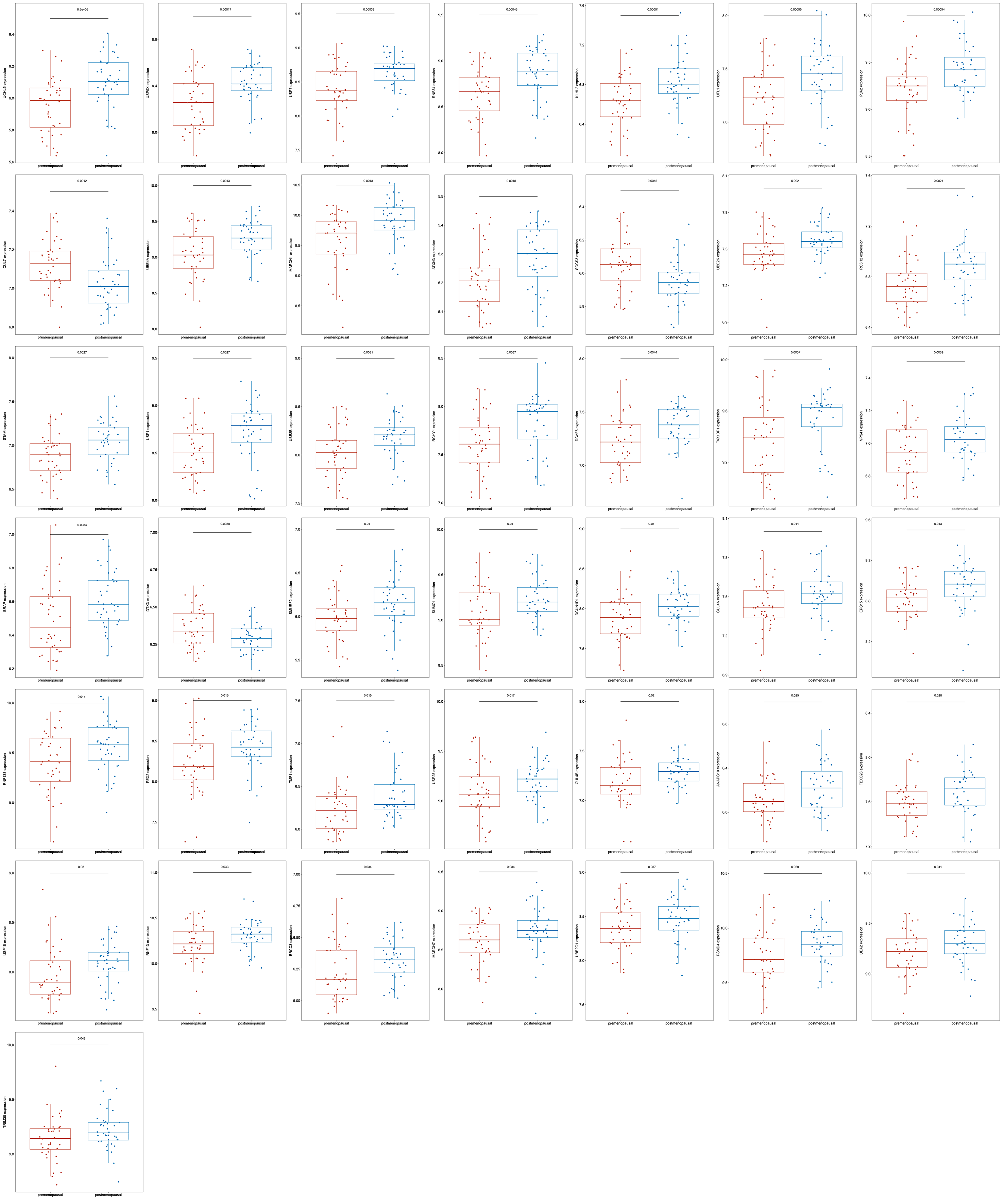

Supplement: Supplementary file 2 [file Image2.TIF]
